# Challenges, facilitators and barriers to the adoption and use of a web-based national IRD registry: lessons learned from the IRD-PT registry

**DOI:** 10.1186/s13023-022-02489-1

**Published:** 2022-08-26

**Authors:** João Pedro Marques, Sara Vaz-Pereira, José Costa, Ana Marta, José Henriques, Rufino Silva

**Affiliations:** 1 Ophthalmology Unit, Centro de Responsabilidade Integrado em Oftalmologia (CRIO), Centro Hospitalar e Universitário de Coimbra (CHUC), Praceta Prof. Mota Pinto, 3000-075 Coimbra, Portugal; 2 Clinical Academic Center of Coimbra (CACC), Coimbra, Portugal; 3 Faculty of Medicine, University Clinic of Ophthalmology, University of Coimbra (FMUC), Coimbra, Portugal; 4 Department of Ophthalmology, Centro Hospitalar Universitário de Lisboa Norte (CHULN), Lisbon, Portugal; 5 Department of Ophthalmology, Faculdade de Medicina, Universidade de Lisboa (FMUL), Lisbon, Portugal; 6 Ophthalmology Unit, Hospital de Braga (HB), Braga, Portugal; 7 Ophthalmology Unit, Centro Hospitalar Universitário do Porto (CHUPorto), Porto, Portugal; 8 Instituto Ciências Biomédicas Abel Salazar (ICBAS), Porto, Portugal; 9 Instituto de Oftalmologia Dr. Gama Pinto (IOGP), Lisbon, Portugal; 10 Instituto de Retina de Lisboa (IRL), Lisbon, Portugal

**Keywords:** Registries, Retinal degeneration(s), Rare disease(s), Ophthalmic genetics, User engagement

## Abstract

Rare disease registries increase research accessibility for patients, while providing clinicians/investigators with a coherent data ecosystem necessary to boost research and patient care. The IRD-PT registry is a national, web-based, interoperable registry for inherited retinal degenerations (IRDs) designed to generate scientific knowledge and collect high-quality data on the epidemiology, genomic landscape and natural history of IRDs in Portugal. In two years, the number of enrolled patients almost doubled (537 to 1060). Still, the registry has a lower-than-expected adoption rate, with only 4 centers across Portugal actively enrolling patients. This highlights a strong need to understand factors that may be hindering the registry’s nationwide adoption. The purpose of this manuscript is to analyze challenges, facilitators and barriers to the adoption and use of the IRD-PT registry, and to discuss avenues for improvement, focusing on keeping the registry sustainable in the long run. We believe that this exercise may help other rare disease registries to improve user adherence and engagement, ultimately contributing to develop more sustainable and successful registries in the field.

## Background

The development of multicenter patient registries promotes the generation of scientific knowledge by using real-world data. Whilst rare diseases gain visibility as a public health priority and the marketplace expands, acknowledgement of the importance of building collaborative relationships in rare disease research increases [1]. Having data stored in a registry will reflect local workloads and burdens of disease, so as to support facilities’ needs for appropriate allocation of human and infrastructure resources. Rare disease registries increase research accessibility for patients, while providing clinicians/investigators with a coherent data ecosystem necessary to boost research and patient care. Inherited retinal dystrophies/degenerations (IRDs) are a clinically and genetically heterogenous group of diseases with an estimated prevalence of 1 in 3000 individuals [2]. Despite some common ground, genetic profiles vary considerably among regions and ethnic groups, thus highlighting the importance of obtaining reference population-based data. The IRD-PT registry [3] is a national, web-based, interoperable registry for IRDs designed to generate scientific knowledge and collect high-quality data on the epidemiology, genomic landscape and natural history of IRDs in Portugal. The IRD-PT pre-launched in mid-2019 at *Centro Hospitalar Universitário de Coimbra* (CHUC), the only Portuguese health care provider (HCP) that integrates the European Reference Network for Rare Eye Diseases (ERN-EYE) and the largest IRD reference center in Portugal. Testing the registry in a pilot center before its national debut aimed to identify possible problems during data completion, test the time spent in data entry, and detect information gaps or system inaccuracies. The registry proved fully functional and easy to use. As of April 30th 2022, data from 1060 IRD patients is now included in the registry, approximately twice the number of patients enrolled in April 2020 (n = 537) [3]. Considering the Portuguese population (~ 10 million inhabitants), this number corresponds to roughly 1/3 of the total estimated cases of IRDs in Portugal. Other than CHUC (n = 890 patients included), 3 centers are actively enrolling patients in the registry: *Centro Hospitalar Universitário Lisboa Norte* (CHULN, n = 58 patients included), *Hospital de Braga* (HB, n = 58 patients included) and *Centro Hospitalar Universitário do Porto* (CHUP, n = 54 patients included). While the numbers are satisfactory, the registry has a lower-than-expected adoption rate. Based on user feedback and peer-to-peer discussion, we decided to conduct a critical analysis to understand factors that may be hindering the registry’s nationwide adoption. Thus, the purpose of this manuscript is to analyze challenges, facilitators and barriers to the adoption and use of the IRD-PT registry, and to discuss avenues for improvement, focusing on keeping the registry sustainable in the long run.

## Challenges, facilitators and barriers

In a rapidly evolving field such as IRDs, there is an urge to improve quality of care to conform to standards. An IRD patient registry helps align IRD specialists from different departments and facilities towards one uniform format of data recording. Yet, there are challenges to embrace and barriers to overcome when adopting a registry. Recognizing and understanding the nature of such challenges and barriers is imperative to be well equipped to devise strategies to overcome them.

Lack of time is probably the most significant hindrance to the adoption and use of a registry. Ophthalmologists have limited time with patients during office visits, and electronic health record (EHR) use requires a substantial portion of that time, therefore affecting productivity [4, 5]. To promote acceptance and use, registries must be able to adequately interface with other IT systems and exchange information [6]. Unfortunately, there are several EHR vendors operating in Portugal, each with different data capturing systems. Due to the lack of structure and standardization of EHR data, most registries still operate in a mixed data collection environment with continued dependence on manual data entry through clinical chart abstraction [7]. Thus, improvement in semantic interoperability between registries and source data systems is highly needed. The IRD-PT registry [3] allows EHR third-party applications with structured information to deliver their data directly to specific subfields of the registry, thus enabling a quick fill-in process and promoting workplace efficiency. Additionally, by adopting a minimum mandatory data set, the IRD-PT registry [3] helps reduce the proportion of missing data on a patient file and improve the care process by providing guidance and prompt on necessary elements of the clinical history. Still, the balance between record completeness and user burden is not easy to achieve. On the one hand, end-user engagement increases when mandatory data is kept to a minimum. On the other hand, this means that there might be incomplete information/missing data for some enrolled subjects regarding unanswered, non-mandatory fields. We are currently testing data mining from EHR as a strategy to decrease the dependence on manual data entry.

Individual attitudes and beliefs have been reported to act as both facilitators and barriers to implementation and acceptance of e-health systems across all e-health domains [6]. Interest in technology, perceived usefulness and motivation are positive attitudes associated with increased acceptance and implementation. Conversely, general resistance to change, distrust in the system, concerns over patient privacy and security being compromised, or doubts that the registry can actually improve patient care, clinical outcomes or quality of practice act as barriers. Many healthcare professionals believe e-health systems disrupt workflows and the delivery of care [8]. A change of mindset is needed at the practice level in order for clinicians to gain value from their registry participation [7]. Demographic factors such as age, education, sex, nationality, and clinical experience may also influence healthcare professionals’ attitudes towards e-health systems [9]. Interestingly, all doctors actively enrolling patients in the IRD-PT registry [3] are ≤ 40 years old. As millennials, their generation is marked by elevated usage of and familiarity with the internet, mobile devices, and social media. Higher technological literacy is likely to potentiate quicker adoption and engagement. Financial incentives may be used as strategies to overcome resistance and stimulate participation [6]. These include financial sponsorship (e.g.: society membership fee reduction or congress fee reduction for adopters), reimbursements for adoption, and pay-for-performance initiatives. Although we believe these interventions may make data introduction more appealing for some users at first, we are not convinced that this is sustainable in the long run. Alternatively, we are working on the integration of the IRD-PT registry [3] with other IRD international registries [Rare Eye Disease Registry (REDgistry) from the ERN-EYE and Fight Inherited Retinal Blindness (FIRB!) registry from the Save Sight Registries project], aiming to motivate users by the possibility to have their name featured in relevant publications or easing access to clinical trials. Interoperability has always been a key issue during the development of the IRD-PT registry [3]. All diagnoses are coded according to the International Statistical Classification of Diseases and Related Health Problems (ICD) 9, 10, 11, and Orphanet Rare Disease Ontology (ORPHA) numbers. Furthermore, genes are coded according to the Ontology of Genes and Genomes (OGG) and Mendelian Inheritance in Man (MIM), and patient signs and symptoms are coded according to the Human Phenotype Ontology (HPO). By resorting to common data elements, core outcome sets, and standardized data structures, the IRD-PT can support the exchange of data across datasets, facilitating its connection to other registries at an international level.

Appropriate, high-quality, and easily available training is a facilitator to the implementation of a registry, whereas it can be considered a barrier when it is non-existent or existent but inadequate [6, 10]. With this in mind, we recently developed short *how-to* videos aiming to explain basic functions of the registry such as: creation of a new patient, retrospective data introduction, new clinic or treatment visit, or data analysis. These videos were made available at the Portuguese Society of Ophthalmology website for all members to access. Additionally, the registry has been advertised in national congresses and meetings and a manuscript detailing its design, development and deployment was published in an open access journal [3].

Complexity factors such as slow system performance, data handling, reliability, unplanned downtime and connectivity issues negatively influence the adoption and use of systems in healthcare settings [6]. Fortunately, this is not the case with the IRD-PT registry [3]. End-users were involved in its design and development, thus selecting IRD specific information for a smooth data capture. Additionally, the platform is user-friendly, web-based (thus available anywhere, including mobile platforms), and is managed by an IT team that provides end-user technical support around the clock.

Blumenthal [7] identified cost as the most significant barrier to the long-term sustainability of clinical registries. As part of the *retina.pt* platform (https://www.retina.com.pt), developed by the Portuguese Retina Study Group (GER, www.ger-portugal.com), the registry receives annual funding from industry stakeholders (Novartis^®^, Bayer^®^, Allergan^®^ and Alimera^®^), making its use available to all members of the Portuguese Society of Ophthalmology at no extra cost. Funding is used for data management activities, IT support, layout improvements and legal support. However, these companies have no proprietary interest in the generated data.

## Avenues for improvement

Despite the high number of enrolled patients, only 4 centers across Portugal have adopted and are currently using the registry. Lack of time, individual attitudes and beliefs and low technological literacy are the most significant challenges and barriers to a nationwide embracement of the registry. Our approach for the future involves making data capture easier and less time-consuming for the users with the development of additional training materials like the how-to videos and the implementation of data mining from EHR to decrease dependence on manual data entry. Additionally, we aim to make the adoption and use of the registry more appealing with the integration in other international IRD registries, and the publication of multicenter studies with data from the registry. We hope that combining these strategies with the existing strengths of the IRD-PT (user-friendly interface, minimum mandatory data set, web-based format, around the clock IT support, and robust funding to ensure long-term sustainability) will attract and fixate new users.

In conclusion, we provide insight into factors whose interplay may lead to improved end-user adoption and engagement in a national IRD patient registry. Sustainability in the long run can only be met by fostering a culture of communication and cooperation between users and adopting realistic strategies to overcome challenges and barriers. We believe the implementation of the above mentioned strategies will make the IRD-PT more functional, pervasive and sustainable. Additionally, we hope that this exercise may help other rare disease registries to improve user adherence and engagement, ultimately contributing to develop more sustainable and successful registries in the field.

## Data Availability

The datasets used and/or analyzed for the current manuscript are available from the corresponding author on reasonable request.
